# Correlation of the Cognitive, Psychomotor and Affective Domains of Professionalism Assessment in Dental Graduates

**DOI:** 10.12669/pjms.42.3.12101

**Published:** 2026-03

**Authors:** Saba Rehman, Usman Mahboob, Attique ur Rehman, Faiza Salman

**Affiliations:** 1 Saba Rehman Department of Medical Education, University College of Medicine and Dentistry, University of Lahore, Pakistan.; 2 Usman Mahboob Professor in Medical Education, Institute of Health Professions Education & Research, Khyber Medical University, Peshawar, Pakistan.; 3 Attique ur Rehman Head of Department, Assistant Professor, Department of Operative Dentistry, Islam Dental College, Sialkot, Pakistan.; 4 Faiza Salman Assistant Professor, Department of Medical and Dental Education, Rashid Latif Dental College, Lahore, Pakistan

**Keywords:** Professionalism, Dental education, Attitude, Cognition, Assessment, Learning domains

## Abstract

**Objective:**

To investigate the correlation between the Situational Judgment Test (SJT), Observed Structured Practical Examination (OSPE), and Multi-Source Feedback (MSF) for the purpose of assessing professionalism across the three learning domains.

**Methodology:**

A correlational study was done to investigate the correlation between the scores of SJT, OSPE, and MSF. The study was conducted at Islam Dental College, Sialkot. Data collection was completed in four months. Census sampling technique was employed. Date was collected through validated tools i.e., SJT, OSPE and MSF (360-degree evaluation). Correlation test was conducted to analyze the correlation relationship among the three variables i.e., scores of SJT, OSPE and MSF scores.

**Results:**

Demographic data showed that sample consists of 29.4% male and 70.6% female participants. There was a statically significant relationship between three domains (P<0.05). The correlation value between SJT and MSF was 0.35, whereas the correlation value between SJT and OSPE was 0.43. The correlation value between MSF and OSPE was 0.57, the strongest among all, indicating a moderate correlation.

**Conclusion:**

The correlation coefficients obtained from the analyses shed light on the degree of alignment between cognitive knowledge, practical skills, and professional behavior. Positive correlation was observed between the SJT, OSPE, and MSF indicating that these assessment methods are capturing the related competencies. Strong performance in one area is likely to reflect the strong performance in the others. It encourages the need for more integrated assessments to link theoretical learning with practical application in a meaningful way.

## INTRODUCTION

Medical Professionalism is a fundamental aspect of healthcare, and it encompasses a range of behaviors and qualities, expected of medical professionals.^1^,^2^ Professionalism in medicine is a multifactorial process comprising individual values, beliefs, and environmental factors. These values manifest in observable behaviors, making it possible to measure professionalism through consistent actions and decisions in various settings. Another critical aspect is acquiring professional knowledge, which includes the necessary knowledge, skills and qualifications.^3^

The complexity of professionalism necessitates a multi-dimensional assessment, considering the cognitive processes, interpersonal skills, and other attributes. Research by Kuang Teck Tay (2020) emphasizes the need to integrate different assessment methods and criteria to capture the full spectrum of professionalism.^4^ Additionally, Wong et al., (2022) in his study highlighted that different assessment methods should be used in medical teaching to reflect the application of knowledge of professionalism in a clinical setting.^5^ Single method of assessment will be limited to only one level of Miller’s pyramid, and hence, assessment of one attribute can leave other characteristics unassessed, making the evaluation of professionalism more complicated.^6^

Professionalism in dental education encompasses a blend of cognitive knowledge, technical skills, and interpersonal qualities.^7^ However, assessing these diverse competencies holistically remains challenging, as each domain-cognitive, psychomotor, and affective-captures unique aspects of professional development.^8^,^7^ A few studies have been reported that use multiple assessment tools. One study employed JSPE-S, SJT, and OSCE for the assessment of empathy while another compared DOPS and MCQ to assess the approaches to learning.^9^,^10^ However, there is little evidence about the correlation among the assessment tools used to assess professionalism, spanning the three learning domains. Understanding how these tools interact and influence one another is crucial for developing well-rounded dental professionals who can seamlessly integrate their knowledge with practical skills and professional behavior in real-world settings.^4^,^11^

The current research aimed to examine whether there is a correlation between assessment tools of professionalism for the cognitive, psychomotor, and affective domains, which would indicate that students effectively integrate learning across the three learning domains i.e., cognitive knowledge with practical and interpersonal skills. The rationale is formulated using Miller’s Pyramid as a framework, understanding how these tools correlate with each other parallel to different levels of competence: knows, knows how levels are assessed through the Situational Judgment Test (SJT), shows how level is assessed via Objective Structured Practical Examination (OSPE), and does level will be assessed through the Multisource Feedback (MSF).^4^

## METHODOLOGY

This research study was conducted at Islam Dental College, Sialkot, affiliated with the University of Health Sciences (UHS).

### Ethical Approval:

The study protocol received full review and approval by two independent ethical review boards**:** (1) Ethical Review Board (ERB) of the University College of Medicine and Dentistry (UCMD) and University of Lahore (UOL), Reg. No. ERC/141123112; Dated: December 12, 2023 under the title ‘Correlation of the Cognitive, Psychomotor, and Affective Domain of Professionalism Assessment in Dental Graduates’. (2) Ethical Review Board (ERB) of Islam Dental College Ref.no: IDC-24-7909; Dated: March 4, 2023.

Data collection was completed in four months, from the month of March 2024 to June 2024. It employed a correlational research design using a quantitative analytical approach to explore the relationships between three assessment tools: SJT, OSPE, and MSF.^12^ Assessment tools were selected based on relevance to the each learning domain, feasibility, practicality and level of learners.^4^ To ensure validity and reliability, validated SJTs were sourced from a prior study conducted on final-year dental students in Pakistan to assess the non-cognitive attributes.^13^ OSPE was constructed following the steps of development of questions using AMEE Guide 81.^14^,^15^ To ensure qualitative content validity, each OSPE station was reviewed by experts to confirm that it measured the relevant psychomotor skills associated with professionalism in dental practice. To ensure both validity and reliability of the MSF form, the version used in this study was adapted from AMEE Guide No. 31.^16^ The assessment process involved collecting ratings from Head of departments and senior faculty members who had completed supervisory workshops and were trained in the use of multisource feedback (MSF). These individual scores were combined to produce an overall MSF score, which was then used for comparison and correlation analyses.

A census sampling technique was used to include all house officers in the study.^12^ All dental house officers who had completed at least six months of house job were involved. House officers who took extended leaves that may have interrupted their house job for a significant period were excluded. House officers with previous extensive experience (e.g., extra internships etc.,) in clinical settings were excluded. Informed consent of candidates was obtained before their inclusion in the study.

### Data Analysis:

Data analysis was performed using SPSS v25 software. Shapiro-Wilk test & Kolmogorov Smirnov test were applied to determine the normality of the data. Pearson’s correlation test with Kruskal-Wallis test of significance was applied to determine the relationships among the three variables i.e., SJT, OSPE, and MSF scores which will depict the scores of assessments of professionalism in three learning domains.^17^

## RESULTS

A total of 40 participants were available for the study. In terms of demographics, the majority of the sample consists of female house officers; there were 12 (29.4%) male and 28 (70.6%) female participants. In general, participants were of a similar age at the time of graduation from final year to house job, the mean age of the participants was calculated as 23.97 + 1.81 years with minimum and maximum values of 22.0 and 28.0. Table-I summarizes the results from tests of normality which show that the data was not considered normally distributed.

**Table-I:** Results of Test of Normality.

	Kolmogorov-Smirnov			Shapiro-Wilk Test		
	Statistics	df	Sig.	Statistics	df	Sig.
SJT	0.26	34	0.00	0.78	34	0.01
MSF	0.17	34	0.01	0.93	34	0.51
OSPE	0.203	34	0.03	0.87	34	0.01

Although the data was not following the normality, Pearson’s correlation test was used to determine the relationship between variables as it the more appropriate and more precise method for assessing the strength when data consists of continuous variables. (Table-II: Results of Correlation).

**Table-II:** Results of Correlation Relationship among the three scores depicting the domains of learning.

Pairs	Pearson Correlation (r)	p-value	Statistical Significance
SJT and MSF	0.35	0.039	Significant (p < 0.05)
SJT and OSPE	0.43	0.011	Significant (p < 0.05)
MSF and OSPE	0.573	0.00	Significant (p < 0.01)

SJT and MSF: (r= 0.35): This shows a weak correlation but is statistically significant because the p-value is less than 0.05 (p=0.039); SJT and OSPE: (r = 0.43): This shows a moderate correlation but is statistically significant, with a p-value below 0.05 (p=0.011); MSF and OSPE: (r = 0.573): This depicts a stronger correlation and is highly statistically significant, with a p-value less than 0.01 (p=0.0).

## DISCUSSION

The principal objective of the present study was to investigate the degree of correlation between the scores of the SJT, OSPE, and MSF to assess professionalism in all learning domains. The results of our analysis provide an insightful answer to this research question. The correlation values suggest that these three assessment methods are related to evaluating the student’s professionalism across three learning domains. The correlation between *SJT and OSPE* indicates that cognitive and psychomotor domains are interconnected to some extent. The correlation is moderate, not strong, which highlights that the difference in scores of assessments may be because the participants show professionalism on SJT involving hypothetical scenarios while in OSPE they are performing their behaviors in a controlled environment.^18^ An overall good performance of students in both knowledge-based and skills-based assessments, may imply that learning experience supports both understanding and application.

A review article by Chang found the correlation coefficients between OSPE and written test measures, including MCQ & short answer tests.^19^ The value was calculated to be around 0.50 but, it is worth noticing that the study involved a scoping review, using different articles, and hence the cohort of students was different. In contrast, our study involved the same cohort of students completing both SJT and OSPE, resulting in a moderate correlation, indicating integration between cognitive and psychomotor domains. In the same vein, Couto found correlations among different types of assessment in the PBL session; formative assessment in PBL, which assesses attitudes; OSPE, which assesses skills, and progress tests which assess the cognitive domain.^20^

The correlation value between *SJT and MSF* assessment was low. The weak relation indicates that, both tools may also be capturing distinct learning domains and the two assessments are likely measuring only a few overlapping aspects of a participant’s abilities in each domain.^21^ This segment of the study needs more research to evaluate the reasoning behind the low values of correlation. The strongest correlation value was observed between the MSF and the OSPE**,** which indicates that individuals who exhibit strong affective qualities are expected to perform well in professional tasks.

Both the MSF assessment and the OSPE evaluate performance, which could indicate that the proficiencies evaluated in the OSPE, under the supervision of a teacher, are closely related to the professional behaviors and communications that are esteemed in everyday professional settings, as reflected in the MSF. These correlation values suggest that these different assessment methods are related, supporting an integrated approach to evaluating student professionalism. Most studies identified a strong correlation between OSPE and other assessment tools like Simulated Patient Scenarios^22^ and traditional oral clinical examination (TOCE) using real patients,^23^ however in the present study OSPE was correlated with performance as well as written assessment tools.

The findings suggest several implications for dental education and assessment policy makers. This study was conducted among house officers who had successfully passed their undergraduate high-stake examinations. Therefore, the findings are true reflection the participants’ knowledge and behaviors instead of examination-related performance. For educators, teachers, and stakeholders, a combination of these assessments can be employed in long-term assessment to gain a more complete understanding of an individual’s abilities, ensuring the consideration of cognitive, practical, and affective qualities. Future studies will correlate these scores with patient satisfaction or complaints as recorded by taking in view the patient’s response. Additionally, the predictive values of SJT for the scores of OSPE and MSF should be assessed to predict if success in one area can often predict success in another, making these assessments powerful when used together.

### Limitations:

One of the main limitations of the study is that this study is limited to one dental college with small sample size, which restricts the cultural context. The specific teaching methods and assessment practices of a single college may make it difficult to apply the results to colleges with diverse educational methods. Furthermore, the study consisted of a single-time assessment. A study assessing long-term assessment employing different assessment tools should be conducted.

## CONCLUSION

A strong cognitive learning base reflects strong psychomotor and affective domains. It implies that students having good knowledge and understanding of professionalism can effectively demonstrate professionalism in controlled as well as real-life environments. In healthcare, the understanding of professionalism and ethics provides a foundation for understanding complex situations, whether in a simulated setting or professional practice. This research can assist medical schools in developing effective programs for teaching, assessing professionalism, and producing professional and compassionate physicians. It not only identifies the areas of improvement in students, but it can also ensure that assessments accurately reflect a trainee’s professional abilities to apply their theoretical knowledge of professionalism into real life.

### Author’s Contribution:

**SR:** Conceived, designed. editing of manuscript, and is responsible for integrity of research.

**AUR:** Did data collection & statistical analysis. Critical Review

**FS:** Critical analysis, manuscript writing.

**UM:** Review and final approval of manuscript.
